# Neuronal Plasticity in the Mushroom Bodies of Winter Bees Is Retained Despite Substantially Advanced Age

**DOI:** 10.1002/dneu.23006

**Published:** 2025-09-27

**Authors:** Nadine Kraft, Wolfgang Rössler, Claudia Groh

**Affiliations:** ^1^ Biocenter, Behavioral Physiology and Sociobiology University of Würzburg Würzburg Germany

**Keywords:** aging, *Apis mellifera*, microglomeruli, mushroom body, structural synaptic plasticity, winter bees

## Abstract

Honeybee (*Apis mellifera*) workers exhibit remarkable behavioral plasticity throughout adult life. In spring and summer, they transition through diverse tasks over a short lifespan of 4–6 weeks. This involves dramatic changes in sensory environment and cognitive demands associated with pronounced structural neuronal plasticity in the mushroom bodies (MBs), high‐order brain centers for sensory integration, learning, and memory. This plasticity manifests as age‐ and experience‐related volume increase in sensory input regions of the MB calyces, accompanied by pruning of projection neuron (PN) boutons in synaptic microcircuits within visual and olfactory compartments. As winter approaches, honeybees suspend brood rearing and foraging activities to survive the cold months by forming a tight, thermoregulated cluster. Unique physiological adaptations enable winter bees to live up to 8 months until a new generation emerges in spring. This extended lifespan occurs during a period of reduced sensory input and high metabolic costs raising the question of how such conditions affect structural neuronal plasticity. Using synapsin immunolabeling and 3D confocal‐microscopy image analyses of MB synaptic neuropils in whole‐mount brains of age‐controlled worker bees, we found that winter bees retain a high degree of neuronal plasticity throughout their lifespan. MB calyces exhibit an initial volume increase followed by a period of stagnation to then undergo another expansion at the onset of spring foraging. While olfactory PN boutons exhibit continuous pruning, visual bouton numbers remain stable during winter. We conclude that winter bees retain comparable neuronal capacities to summer bees, despite strong differences in lifespan, physiological, and environmental conditions.

## 1 Introduction

Winter bees of the Western honeybee (*Apis mellifera*), also termed diutinus bees, represent a distinct physiological and behavioral phenotype of honeybee workers that enables honeybee colonies to survive periods of resource scarcity and low temperatures in winter (Fluri et al. 1982; Seeley and Visscher 1985; Winston 1987; for a review, see Knoll et al. 2020). In contrast to summer worker bees with a typical lifespan of 4–6 weeks, winter bees can live up to 8 months, thus persisting throughout the entire overwintering period (Amdam and Omholt 2002; Fluri et al. 1982; Fukuda and Sekiguchi 1966). The transition from summer to winter bees, which typically occurs during fall, is a gradual process most likely triggered by a combination of environmental factors, such as decreasing day length and temperature combined with resource scarcity, and colony cues including a cessation of brood rearing and changes in pheromonal influences (discussed in Döke et al. 2015; Fluri and Bogdanov 1987; Huang and Robinson 1995; Mattila et al. 2001; Mattila and Otis 2007; Maurizio and Hodges 1950). Winter bees exhibit distinct physiological traits, such as enlarged fat bodies (Fluri and Bogdanov 1987), hypertrophied hypopharyngeal glands (Fluri et al. 1982), elevated levels of vitellogenin (Fluri et al. 1977), and reduced juvenile hormone titers (Fluri et al. 1977; Huang and Robinson 1995), which are presumed to collectively contribute to their enhanced longevity and stress resistance (Amdam and Omholt 2002; Münch and Amdam 2010). These physiological changes are closely linked to behavioral adaptations during wintertime, such as reduced foraging activity and the formation of a thermoregulated winter cluster (Schulz et al. 2002; Southwick 1985). Unlike true hibernation, winter bees do not enter an inactive, dormant state; instead, they form a tight cluster of ∼10,000–20,000 bees and engage in thermoregulation to maintain temperatures between 20 and 30°C in the inner core of the cluster and a minimum temperature of ∼12°C at the outer edges (Fahrenholz et al. 1989; Knoll et al. 2020; Seeley and Visscher 1985; Southwick 1983; Southwick and Heldmaier 1987; Stabentheiner et al. 2003). While it is not uncommon for winter bees to occasionally leave the hive to perform cleaning or defecation flights on days with favorable weather conditions, they usually remain inside the hive throughout the entire winter period, living on their stored food reserves (Southwick 1991).

Between mid‐ and late winter, largely dependent on ambient temperature, but potentially also on flower availability (discussed in Döke et al. 2015), brood rearing is gradually resumed (Nürnberger et al. 2018; Seeley and Visscher 1985). As a consequence, the cluster raises its core temperature to a constant level of 33–35°C to provide optimal rearing conditions for a new generation of worker bees, which will gradually replace the aged winter bees in the upcoming spring period (Groh et al. 2004; Moeller 1977; Owens 1971). Interestingly, as soon as overwintered winter bees engage in foraging tasks in spring, their juvenile hormone titers increase, while vitellogenin titers and hypopharyngeal gland weight decrease, resembling the physiological state of a foraging summer bee (Fluri et al. 1982). Thus, it seems likely that winter and summer bees, despite their significant differences in age and life history, possess similar behavioral capacities and plasticity even though the timescale of events is largely extended in winter bees. A typical summer bee progresses through a variety of different tasks during its rather short lifespan of 4–6 weeks. In the first 2–3 weeks after adult emergence, a summer worker bee performs nursing and cleaning tasks in a mostly olfactory‐dominated environment inside the dark hive (Robinson 1992; Winston 1987). Afterward, workers gradually shift to outdoor tasks, and forage for nectar, pollen, and water, which comprises an entirely new set of challenges, demands, and drastic changes in the sensory environment (Robinson 1992).

**FIGURE 1:**
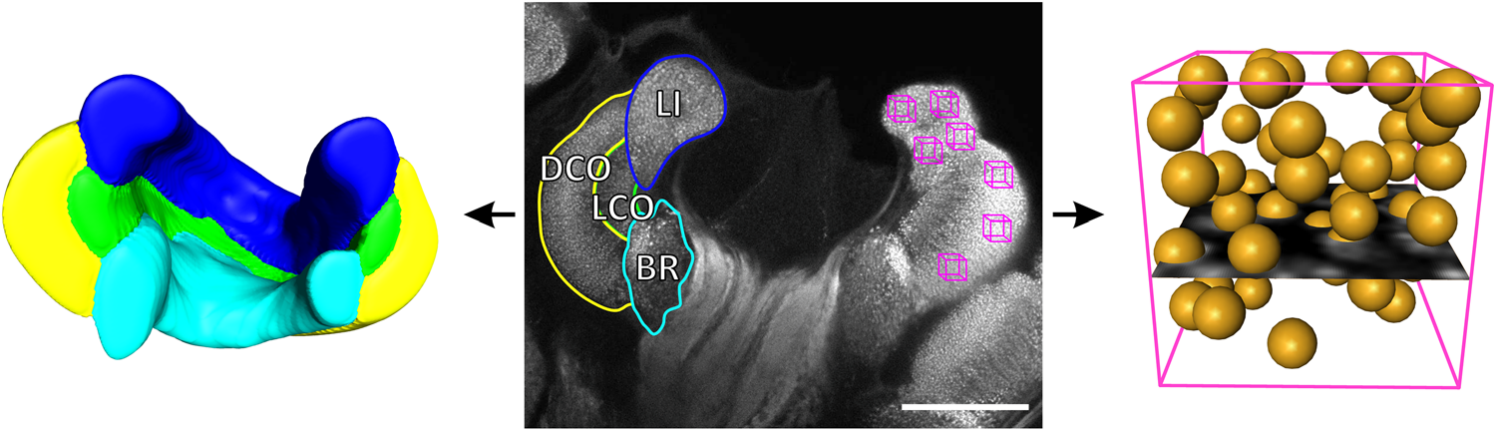
Quantification of mushroom‐body (MB) calyx volume and projection neuron (PN) boutons in winter bees. Middle: Frontal view of a synapsin immunolabeled MB calyx in a whole‐mount brain of a winter worker bee showing the different calyx compartments lip (LI, blue), dense collar (DCO, yellow), loose collar (LCO, green), and basal ring (BR, cyan). Magenta cubes represent the regions of interest in LI and DCO used for PN bouton quantification (not to scale). Left: 3D reconstruction of a medial MB calyx cut in a medial plane to reveal the individual calyx compartments. Color coding is consistent with the middle image. Right: A 1000‐µm^3^ cube representing a region of interest for 3D PN bouton quantification in the DCO. All synapsin immunolabeled PN boutons within the volume are counted by placing landmarks (yellow spheres) throughout a stack of 21 images recorded at 63× magnification. A single optical section of the image stack is shown in the center of the cube. Scale bar = 100 µm.

This marked interior‐exterior transition in summer bees is associated with significant structural changes in higher brain regions, such as the mushroom bodies (MBs)—centers for sensory integration, learning, and memory formation (e.g., Fahrbach 2006; Heisenberg 1998; Menzel 1999; for reviews, see Fahrbach and Van Nest 2016; Groh and Rössler 2020). Their main input region, the MB calyx, undergoes a significant volume expansion throughout adult life, which is mainly caused by dendritic outgrowth of MB‐intrinsic Kenyon cells (KCs; e.g., Durst et al. 1994; Farris et al. 2001; Groh et al. 2012). An initial volume increase already occurs within the first week of adult life (Fahrbach et al. 1998; Muenz et al. 2015) and was also observed in other social insect species, such as ants (Yilmaz et al. 2016) and bumblebees (Jones et al. 2013; Kraft et al. 2019; Riveros and Gronenberg 2010). It is presumed to be an experience‐independent anticipatory plasticity as part of an internal program to prepare workers for upcoming changes in tasks and demands (Fahrbach et al. 1998; for a review, see Groh and Rössler 2020). Later in life, further increases in MB‐calyx volume are mainly driven by the accumulation of experience during foraging and are thus highly dependent on the individual's actions and surroundings (Farris et al. 2001; Withers et al. 2008). In the same line, during volumetric changes, synaptic microcircuits (microglomeruli, MG) within the MB calyces undergo significant structural reorganizations (for reviews, see Fahrbach and Van Nest 2016; Groh and Rössler 2020). Each MG consists of a single presynaptic projection neuron (PN) bouton forming synaptic contacts with many postsynaptic profiles, mainly f‐actin‐rich dendritic specializations of MB KCs (Fahrbach 2006; Frambach et al. 2004; Groh et al. 2006, 2004, 2012). MG are found throughout the entire MB calyx but are spatially segregated in distinct compartments of the calyx depending on their type of primary sensory input: the olfactory innervated lip, the visually innervated collar, and the bimodal basal ring receiving information from both visual and olfactory PNs (Gronenberg 2001; Figure 1). Correlating with age‐ and task‐related behavioral maturation, honeybee MG numbers were found to decrease in both the lip and the densely innervated outer region of the collar (dense collar; Groh et al. 2012; Muenz et al. 2015). An analysis at the ultrastructural level revealed that individual PN boutons are reorganized after bouton pruning, most likely during the switch from nursing to foraging tasks (Groh et al. 2012). Individual boutons increase in size and comprise a higher number of active zones and postsynaptic partners per active zone, leading to an overall increase of KC connectivity and the resulting synaptic divergence of PN boutons in the MB calyx in foragers compared to freshly emerged summer bees (Groh et al. 2012). The study by Scholl et al. (2014) revealed that PN bouton pruning in the dense collar can be precociously induced in dark‐reared worker bees by exposure to controlled light pulses, suggesting that PN bouton pruning during the transition to outdoor tasks, at least partly, is a consequence of non‐associative sensory experience. The exposure to a sensory impoverished environment and associative olfactory learning and long‐term memory formation, on the other hand, promote an increase in olfactory PN bouton numbers (Cabirol et al. 2017; Hourcade et al. 2010). Altogether, these findings highlight that the sensory environment, individual experience, and behavioral maturation have a strong impact on the dynamics of the neuronal architecture in the MB calyces, most likely to provide optimal adaptations and neuronal capacities for the current demands (discussed in Groh and Rössler 2020).

As most of the research on neuronal plasticity, so far, has only focused on short‐lived summer bees, we were interested in investigating the neuronal architecture in the MB calyces of long‐lived winter bees. Not only do they spend an extended amount of time in a dark environment combined with high energy demands according to cold and reduced behavioral task diversity, but they also surpass the lifespan of a summer bee by a considerable range. This opens exciting perspectives for investigating consequences of advanced age on structural neuronal plasticity. To examine this, we sampled winter bees directly from the hive at different time points during winter until springtime followed by synapsin‐immunolabeling–based three‐dimensional (3D) quantifications of MB‐calyx volume together with analyses of densities and absolute numbers of MG in olfactory and visual compartments of the calyx.

## 2 Materials and Methods

### 2.1 Bee Rearing and Age Cohorts

In October 2020, when brood production had almost ceased, a brood comb with sealed worker brood was taken from a honeybee (*Apis mellifera carnica*) hive of the institutional apiary of the Biocenter, University of Würzburg, Germany, transferred to an incubator (HPP 110, Memmert GmbH & Co. KG, Schwabach, Germany) and reared at 34.5°C and 55% relative humidity in constant darkness. Every day around noon, we checked the comb for freshly emerged bees. Over the course of 2 weeks, we individually marked ∼900 freshly emerged bees (≤24 h) under red light by gluing numbered plates (Opalith tags; Heinrich Holtermann KG, Brockel, Germany) onto their thorax. Marked bees were then returned to their colony. One group of bees was directly dissected for further immunohistochemical procedures after emergence, the other groups were sampled from the colony at the age of 2, 4, and 6 months. While 2‐ and 4‐month‐old workers were collected from inside the hive, the 6‐month‐old bees were collected in front of the hive as defined pollen foragers to ensure that they had actively engaged in foraging tasks.

### 2.2 Neuroanatomical Procedures

For dissection, we anesthetized the bees on ice, removed the heads, and fixed the heads in dental wax (Modelling wax; Dentsply International, York, PA, USA). We covered the heads with ice‐cold bee ringer solution (130 mM NaCl, 5 mM KCl, 4 mM MgCl_2_, 5 mM CaCl_2_, 15 mM Hepes, 25 mM Glucose, 160 mM Sucrose; pH 7.2) and cut a window between antennae, complex eyes, and ocelli. Then, we removed trachea and glandular tissue, extracted the brains and immediately fixed them in ice‐cold physiological saline (PBS; 137 mM NaCl, 2.7 mM KCl, 8 mM Na_2_HPO_4_, 1.4 mM KH_2_PO_4_; pH 7.2) with 4% formaldehyde (FA; methanol free, Cat. No. 28908; Fischer Scientific, Schwerte, Germany) overnight at 4°C on a shaker. The following day, we washed the brains in PBS (3×10 min) and cleaned them of excess tissue. Afterward, the brains were permeabilized in PBS with Triton‐X 100 (PBST; AppliChem, Darmstadt, Germany; 1×10 min 2% PBST, 2×10 min 0.5% PBST) and pre‐incubated with 2% normal goat serum (NGS; RRID:AB_2336990, Cat. No. 005‐000‐121; Jackson ImmunoResearch Laboratories Inc., West Grove, PA, USA) in 0.5% PBST for 1 h at room temperature. Then, we incubated the brains with a primary antibody against the *Drosophila* synaptic vesicle‐associated protein synapsin (3c11; 1:50 in 0.5% PBST + 2% NGS; RRID: AB_528479, kindly provided by E. Buchner, University of Würzburg, Germany; Klagges et al. 1996) for 4 days at 4°C on a shaker. After washing in PBS (5×10 min), we incubated the brains in the secondary antibody CF633 goat@mouse (RRID:AB_10582886, Cat. No. 20121; Biotium, Fremont, CA, USA; 1:250 in PBS) for 2–3 days at 4°C on a shaker. Another washing cycle (5×10 min in PBS) was followed by post‐fixation in 4% FA in PBS overnight at 4°C. After rinsing in PBS (5×10 min), the brains were dehydrated in an ascending ethanol series (30%, 50%, 70%, 90%, 95%, 2×100%, 10 min each), cleared in methyl salicylate, and mounted in methyl salicylate in custom‐made aluminum slides.

### 2.3 Image Acquisition and Data Analysis

For image acquisition, we used a Leica TCS SP8 MP confocal laser scanning microscope (Leica Microsystems AG, Wetzlar, Germany) with a 638‐nm diode laser (Coherent, Santa Clara, CA, USA) and internal photomultipliers for signal detection. Images were recorded at a resolution of 1024×1024 pixels and with a frame average of 3. For volumetric analyses, we recorded an image stack of an entire medial MB calyx per brain with either a 20× multi‐immersion objective (HC PL APO 20×/0.75 IMM CORR CS2), or a 10× water‐immersed objective (HC APO L 10×/0.30 W U‐V‐I) with a varying digital zoom and a z‐step size of 5 µm. Detailed scans of lip and dense collar for PN bouton quantification were taken with a 63× glycerol‐immersed objective (HC PL APO 63×/1.30 Glyc CORR CS2) and a 2× digital zoom. Each stack comprised 21 images in steps of 0.5 µm.

All scans were analyzed with the 3D software Amira (version 6.4; Thermo Fisher Scientific, Waltham, MA, USA). In the complete calyx scans, we 3D‐reconstructed the lip, dense and loose collar, and the basal ring using manual segmentation and interpolation (Figure 1, left). This enabled selective volumetric measurements of all calyx regions as well as 3D visualization of the entire calyx. For PN bouton quantification, we cropped defined regions of interest in both lip and dense collar (see boxes in magenta in Figure 1, middle) to a size of 10×10×10 µm and manually counted all PN boutons within these boxes using the landmark function of Amira (Figure 1, right). Later, we calculated the mean over all four boxes in the lip and all three boxes in the dense collar and estimated the total number of PN boutons by extrapolating the bouton density to the volume of the respective calyx region.

### 2.4 Statistics

All statistical tests were performed using the software RStudio (version 2023.06.0; R Core Team 2023). Datasets were tested for normal distribution using a Shapiro–Wilk normality test and for variance homogeneity using Levene's test for homogeneity of variance of the “car” package (Fox and Weisberg 2019). To analyze the effect of age on the volume of the lip and dense collar, as well as the density and absolute number of PN boutons in both regions, we used a one‐way analysis of variance followed by Tukey multiple comparisons of means post hoc test to analyze effects between the different age groups. Sample sizes were *n* = 17 in freshly emerged bees, *n* = 15 in 2‐month‐old bees, *n* = 16 in 4‐month‐old bees, and *n* = 14 in 6‐month‐old bees. Corresponding violin plots were created using the package ggplot2 (Wickham 2016). Graphs for Figure 5 were created in Microsoft Excel (version 2016; Microsoft Corporation, Redmond, WA, USA). All graphs and figures were edited and annotated in CorelDRAW (version 2022; Alludo, Ottawa, Canada).

## 3 Results

**FIGURE 2:**
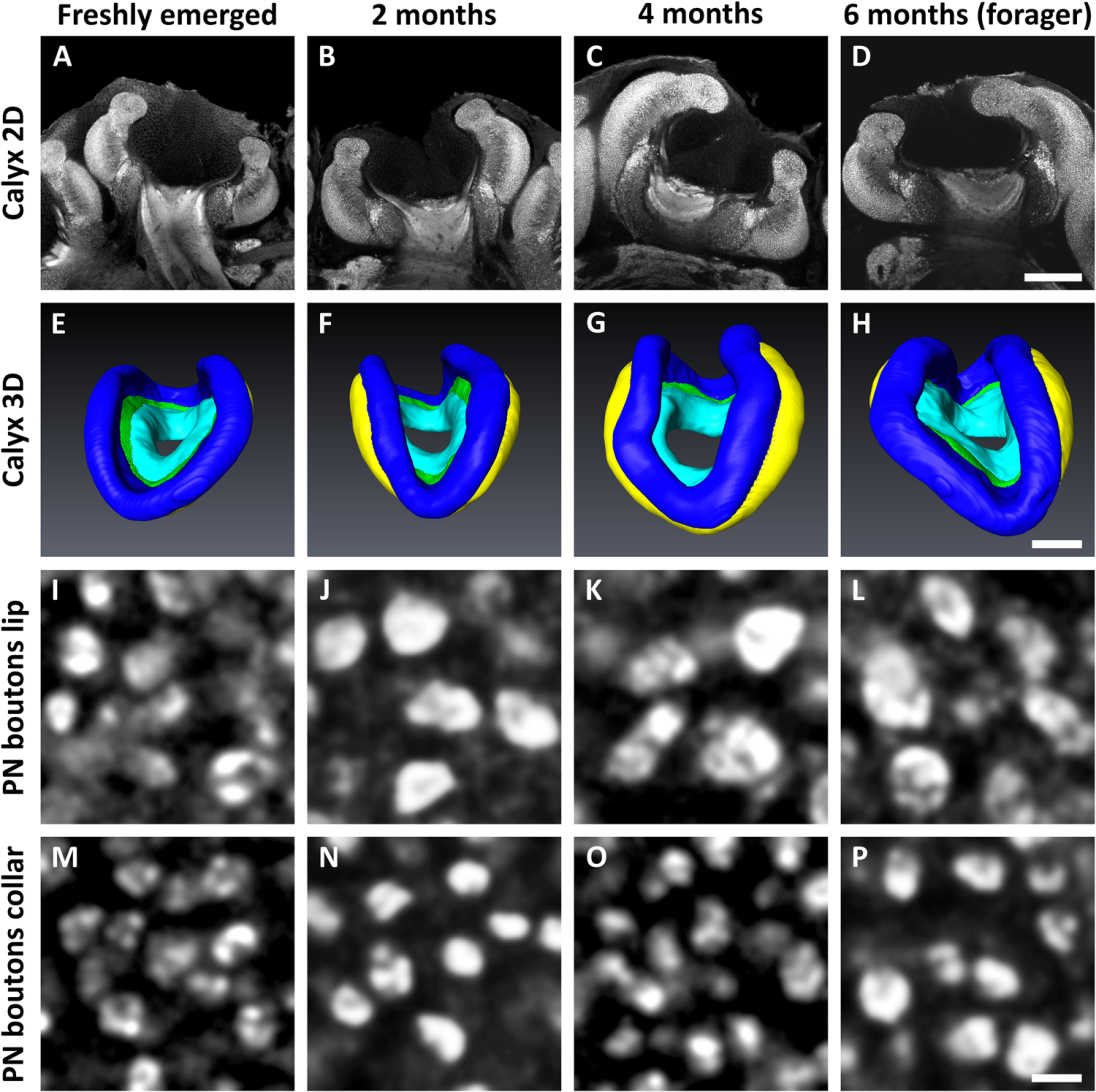
Exemplary visualization of age‐related structural and synaptic plasticity in the MB calyces of winter bees. (A–D) Medial optical sections of synapsin immunolabeled MB calyces of freshly emerged, 2‐, 4‐, and 6‐month‐old winter bees showing the substantial volume increase throughout the overwintering period. (E–H) 3D reconstructions of the MB calyces shown in (A–D). Colors represent the different calyx compartments: lip (blue), dense collar (yellow), loose collar (green), and basal ring (cyan). (I–P) Single optical sections from the lip (I–L) and dense collar region (M–P) showing synapsin immunolabeled PN boutons. In both regions, PN boutons appear to increase in diameter over a 6‐month period. All images per column are sampled from the same MB calyces, respectively. Scale bars = 100 µm in (A–H) and 2 µm in (I–P).

**FIGURE 3:**
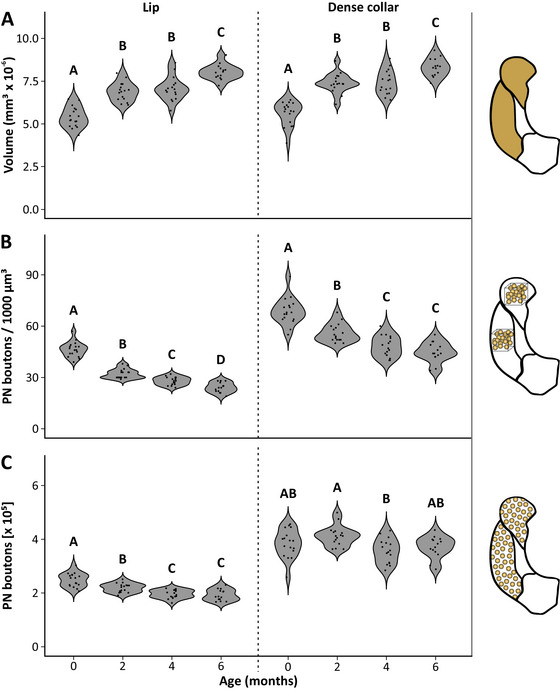
Age‐related structural and synaptic plasticity in the MB calyces of winter bees. (A) Volume of lip (left) and dense collar (right). In both calyx compartments, the volume significantly increases within the first 2 months. Between 2 and 4 months, volumes remain stable but undergo another increase in spring after the onset of foraging. (B) Density of PN boutons in lip (left) and dense collar (right). PN boutons in the lip region exhibit a continuous decrease in density throughout the 6‐month period. A similar decrease is found in the dense collar, although there are no further changes after the age of 4 months. (C) The absolute number of PN boutons in lip (left) and dense collar (right) was determined by extrapolating the bouton density to the volume of each calyx region. Between adult emergence and 4 months, boutons in the lip significantly decrease, indicating synaptic pruning. Bouton numbers in the dense collar are more ambiguous and show no clear trend. Black dots inside the violin plots represent single data points. Capital letters depict statistical differences. Sample size: *n* = 17 in freshly emerged bees, *n* = 15 in 2‐month‐old bees, *n* = 16 in 4‐month‐old bees, and *n* = 14 in 6‐month‐old bees.

**FIGURE 4:**
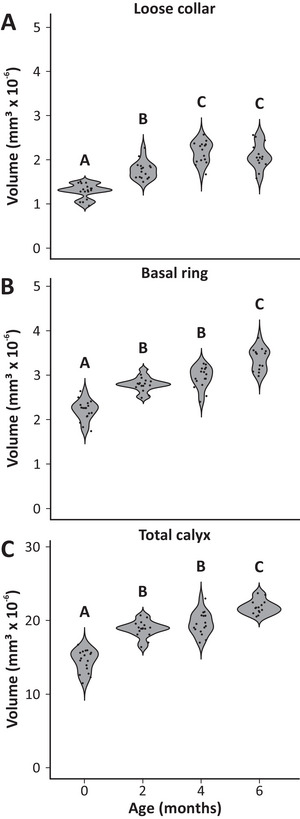
Age‐related structural plasticity in the MB calyces of winter bees. (A) Within the first 4 months of life, the volume of the loose collar region of winter bees continuously increases but shows no further change afterward. (B) The basal ring exhibits a significant volume increase within the first 2 months, remains stable between 2 and 4 months, and exhibits another volume increase with the onset of foraging after 6 months. (C) The volume of the entire MB calyx aligns with the changes observed for the basal ring, as well as the lip and dense collar (Figure 3): An initial volume increase is followed by a period of stability during winter, but with the onset of foraging between 4 and 6 months, the entire calyx undergoes another significant expansion. Black dots inside the violin plots represent single data points. Capital letters depict statistical differences. Sample size: *n* = 17 in freshly emerged bees, *n* = 15 in 2‐month‐old bees, *n* = 16 in 4‐month‐old bees, and *n* = 14 in 6‐month‐old bees.

During the timespan of 6 months between adult emergence in fall and foraging onset in the next spring, the MB calyces in winter bee workers underwent substantial structural changes. Most prominently, we observed a significant volume increase in all MB‐calyx compartments (Lip: *F*(3,59) = 54.51, *p* < 0.001; dense collar: *F*(3,59) = 51.13, *p* < 0.001; loose collar: *F*(3,59) = 50.30, *p* < 0.001; basal ring: *F*(3,59) = 72.03, *p* < 0.001; total calyx: *F*(3,59) = 84.74, *p* < 0.001; Figures 2, 3, 4). In the MB lip and dense collar, an initial volume increase occurred within the first 2 months of adult life (Lip: 0 vs. 2 months: *p* < 0.001; dense collar: 0 vs. 2 months: *p* < 0.001; Figures 2A–H and 3A). Between 2 and 4 months, that is, during the core winter time, the volume remained constant in both regions but underwent another significant expansion upon the beginning of spring and foraging onset (Lip: 2 vs. 4 months: *p* = 0.898; 4 vs. 6 months: *p* < 0.001; dense collar: 2 vs. 4 months: *p* = 0.999; 4 vs. 6 months: *p* = 0.002; Figures 2A–H and 3A). Altogether, the total increase in volume over the 6 months equals to about 49% in the lip and 48% in the dense collar region. While the volume increase of the basal ring was in a similar range compared to the lip and dense collar, the loose collar region (a region with a high proportion of neurites, but fewer MG) showed no further volume increase after 4 months (Basal ring: 0 vs. 2 months: *p* < 0.001; 2 vs. 4 months: *p* = 0.227; 4 vs. 6 months: *p* < 0.001; loose collar: 0 vs. 2 months: *p* < 0.001; 2 vs. 4 months: *p* < 0.001; 4 vs. 6 months: *p* = 0.684; Figure 4A,B). Since the initial volume increase during winter season was similarly pronounced in all four regions of the calyx, this was also reflected in the total MB‐calyx volume with significant increases within the first 2 months and after 4 months of adult life (0 vs. 2 months: *p* < 0.001; 2 vs. 4 months: *p* = 0.397; 4 vs. 6 months: *p* < 0.001; Figure 4C).

The density of synapsin‐immunoreactive (IR) PN boutons was quantified within cubical volumes of 1000 µm^3^ in the lip and dense collar region. Visual inspection of the anti‐synapsin immunolabeled tissue suggested that PN boutons in both calyx regions were rather polymorphic in freshly emerged bees but assumed a more round and distinct shape within the first 2 months of adult life and, most conspicuously in the lip region, considerably increased individual diameters (Figure 2I–P). Furthermore, the density of PN boutons appeared to decrease over the timespan of 6 months, which was confirmed by our quantitative measurements (Lip: *F*(3,59) = 145.80, *p* < 0.001; dense collar: *F*(3,59) = 44.40, *p* < 0.001; Figure 3B). In the lip region, we observed a substantial decrease by almost 70% in the density of PN boutons within the first 2 months (*p* < 0.001). Throughout the remaining 4 months, the density further decreased, albeit to a lesser extent than the initial decrease (2 vs. 4 months: *p* = 0.002; 4 vs. 6 months: *p* = 0.010). The dense collar region showed a similar age‐related decrease in PN bouton density (0 vs. 2 months: *p* < 0.001; 2 vs. 4 months: *p* = 0.006). However, no significant change was found between 4 months and 6 months (*p* = 0.451; Figure 3B).

The absolute number of PN boutons was determined by extrapolating the PN bouton densities to the volume of the respective region. In the lip region, this rendered a significant decrease in PN bouton numbers between adult emergence, 2 months, and 4 months (*F*(3,59) = 26.69, *p* < 0.001; 0 vs. 2 months: *p* < 0.001; 2 vs. 4 months: *p* = 0.008; 4 vs. 6 months: *p* = 0.970; Figure 3C). The effects in the dense collar were more ambiguous (*F*(3,59) = 4.511, *p* = 0.006). Four‐month‐old winter bees had significantly fewer PN boutons than 2‐month‐old bees (*p* = 0.004; Figure 3C). However, none of these groups significantly differed from the other age groups, so no clear trend can be derived from the results in the MB‐calyx collar.

## 4 Discussion

Our study demonstrates that the MB calyces of winter honeybee workers undergo significant structural reorganization over a 6‐month overwintering period. While all MB‐calyx compartments exhibited continuous volume expansion, the density of PN boutons decreased in a comparable range in both the olfactory innervated MB lip and the visually innervated MB dense collar region. Extrapolated bouton counts revealed a slight decline of total PN bouton numbers in the lip region with increasing age, but numbers remained stable in the dense collar region. At first glance, these results mirror the general trend of age‐related structural changes observed in short‐lived summer bee workers. However, the magnitude and timescale of these changes differ substantially between the two worker phenotypes, thus requiring a closer consideration of the underlying plasticity patterns.

### 4.1 Patterns of Structural Plasticity in the MB Calyces

The most striking observation was a uniform volume increase across all four calyx compartments during an initial period of 2 months in winter bees (Figures 3A and 4). Such a volume expansion during adult maturation appears to be a conserved trait among the worker caste in social hymenopterans (Gronenberg et al. 1996; Kraft et al. 2019; Kühn‐Bühlmann and Wehner 2006; Muenz et al. 2015; O'Donnell et al. 2004; Yilmaz et al. 2016; for reviews, see Fahrbach and Dobrin 2009; Groh and Rössler 2020). Notably, MB volume changes are often most pronounced when comparing foragers to in‐hive workers (e.g., Durst et al. 1994; Groh et al. 2012; Ismail et al. 2006; Withers et al. 1993). Since age and behavioral status of workers are tightly linked in most of the studied species due to an age‐related polyethism, both factors can be considered potential determinants of structural plasticity. Using experimental manipulations, several studies provided evidence that the MB volume increase in honeybee workers is indeed part of an age‐related developmental process, as it also occurred despite sensory deprivation, social isolation, and in bees prevented from foraging (Durst et al. 1994; Fahrbach et al. 1998; Withers et al. 1995). This suggests an internal, experience‐independent developmental program that pre‐adapts workers for future demands, such as the upcoming transition to foraging and respective changes in sensory environment. Consistent with this, bumblebee workers, which exhibit a much faster behavioral maturation due to a lack of temporal polyethism, exhibit significant MB‐calyx growth within the first 3 days after adult eclosion, most likely facilitating the early onset of foraging (Kraft et al. 2019). Several studies showed that MB plasticity does not cease after the initial age‐related expansion but rather represents a persistent process throughout adult life. Foraging, for instance, was shown to trigger additional MB growth in honeybees (Farris et al. 2001; Ismail et al. 2006). However, only bees with at least 2 weeks of foraging experience showed significant increases in MB volume compared to less experienced foragers. This suggests that foraging‐associated experience not only acts as a modulator of MB plasticity but also has a cumulative property.

As these innate age‐related and foraging experience‐related increases in MB‐calyx volume are primarily driven by the extensive outgrowth of MB‐intrinsic KC dendrites (Farris et al. 2001; Muenz et al. 2015), a concurrent decrease in the density of presynaptic PN boutons occurs due to spatial displacement of the boutons (Groh et al. 2012; Muenz et al. 2015; discussed in Groh and Rössler 2020). Simultaneously, PN boutons may also undergo pruning, for example, triggered by initial sensory exposure to light (Muenz et al. 2015; Scholl et al. 2014). In contrast, associative olfactory learning and long‐term memory formation can promote the formation of new PN boutons irrespective of volumetric changes (Hourcade et al. 2010). All this suggests that structural plasticity in the MB calyces is a highly dynamic and multilayered process. While KC dendrites continuously expand their network providing the substrate for new synaptic connections, PN boutons are dynamically formed or reduced in response to non‐associative and associative sensory experience and substantially reorganized in response to age and experience (Groh et al. 2012; discussed in Groh and Rössler 2020). Rather than resulting from isolated effects, the plasticity and resulting cognitive capabilities of the MBs are thus fundamentally shaped by the dynamic interplay between PN boutons and KC dendrites. Our methods allowed the examination of overall volumes together with PN bouton densities and numbers in parallel. For better clarity, we discuss them separately within the framework of overwintering.

### 4.2 Analysis of MB Plasticity Patterns of Winter and Summer Bees

When comparing our data with the literature, it becomes clear that winter bees exhibit a similar developmental trajectory compared to summer bees, albeit over a significantly extended timespan. The marked increase in MB‐calyx volume observed during the first 2 months in winter bees likely reflects the early, experience‐independent MB growth typically seen in summer bees (Fahrbach et al. 1998; Muenz et al. 2015; Figure 3A). Although prior studies employed varying time points for the sampling of bees, the magnitude of the initial volume increase remains remarkably consistent. In our study, we observed an approximate 29% growth of the entire MB calyx within the first 2 months, aligning closely with the ∼27% increase reported during the first week of adult life by Muenz et al. (2015). This is also true for the ∼22% MB neuropil expansion documented in two manipulation studies—one following 24 days of in‐hive confinement (Fahrbach et al. 2003), and the other after 7 days of dark‐rearing (Fahrbach et al. 1998). It appears very likely that age cohorts with finer time points in our study would have revealed an onset of volumetric changes at a similarly early time point. Collectively, this strongly suggests that the initial phase of MB expansion represents a conserved and intrinsically regulated developmental process that occurs independently of the individual environment or life history (discussed in Groh and Rössler 2020). Our present results indicate that this pattern persists despite the starkly different ecological contexts of overwintering bees compared to summer bees, which undergo rapid behavioral transitions in a condensed timeframe. It is plausible that the initial neuronal development of the adult MBs, which begins during the late larval and early pupal stages (Farris et al. 1999), is not finalized upon adult eclosion but instead extends into the first days of adult life. This hypothesis is supported by the findings that young worker bees show a weaker performance in associative learning tasks until they are at least 6 days old and that precocious foraging does not occur before the fourth day of adult life (J. Maleszka et al. 2009; R. Maleszka and Helliwell 2001; Ray and Ferneyhough 1999; for a review, see Fahrbach and Robinson 1996) suggesting that the MBs are not fully matured upon the stage of adult eclosion to complete complex tasks.

After the initial volume increase of the MB calyx in this present study, we observed no further change between the age of 2 and 4 months in all analyzed MB‐calyx compartments, except for the loose collar region (Figures 3A and 4). However, after the bees had engaged in foraging in spring, the lip, dense collar, and basal ring underwent another significant volumetric expansion (Figures 3A and 4). This pattern aligns with the experience‐dependent MB growth previously documented in foraging summer bees with extended foraging experience of at least 2 weeks (Farris et al. 2001; Ismail et al. 2006). During the core winter period—characterized by visual deprivation and the inability for foraging—the MB‐calyx volume remains stable, resembling the state of older nurse bees prior to their transition to foraging. Interestingly, these findings contrast with those of Fahrbach et al. (2003), who examined MB plasticity in winter bees. While they also reported a significant MB neuropil volume increase between newly emerged and 24‐day‐old bees, no further changes were detected in overwintered bees. By using behavioral observations and manipulations, they were able to correlate individual foraging experience to the volumetric data. However, they found that bees that had performed foraging trips in fall did not show any significant MB volume differences compared to bees prevented from going outside, regardless of whether they were sampled before or after winter. Notably, in the study by Fahrbach et al. (2003), the overwintered bees were sampled at an age of 146 days in late March, before spring foraging had commenced. In contrast, our spring bees, on average, were 190 days old and sampled as active pollen foragers in late April, when foraging was well underway. This likely allowed them to accumulate sufficient foraging experience to induce a wave of experience‐dependent structural changes in the MBs. As discussed in Fahrbach et al. (2003), the acquisition of forager status represents a critical developmental shift, demanding advanced navigational skills, time and place memory of food sources, multimodal integration of sensory information, and dance communication. This may explain why only highly experienced foragers exhibit MB volume increases—such as shown in Farris et al. (2001) and Ismail et al. (2006)—as prolonged engagement with these cognitive challenges is likely required to drive structural neuroplasticity. It might also explain why sporadic winter foraging (or defecation flights) did not elicit significant MB volume expansion. Occasional flights during times of resource scarcity might not fully activate the molecular neuronal machinery for structural plasticity in the same way as in bees that become fully dedicated to foraging toward the end of their lifespans. Although we did not systematically monitor individual flight behavior, bees in our study were regularly observed leaving the hive on sunny winter days and temperatures well above 10°C, suggesting some pre‐foraging flight experience. Yet, we found no evidence that such sporadic flights influenced MB‐calyx plasticity. An alternative hypothesis by Fahrbach et al. (2003) posits that MB expansion requires both foraging activity and elevated levels of juvenile hormone. Since juvenile hormone titers typically remain low in winter—even during occasional excursions—and only rise again in spring (Fluri et al. 1982; Huang and Robinson 1995), this could explain the absence of volume changes during the winter period.

In addition to volumetric plasticity, our study on the overwintering phase also revealed significant changes at the level of individual PN boutons in the lip and dense collar. We found that PN boutons in the dense collar exhibit an age‐related decrease in density, while extrapolated absolute numbers across the entire calyx volumes remain relatively stable (Figure 3B,C). This indicates that the observed decrease in PN bouton density is not a result of synaptic pruning but rather reflects the expansion in dendritic mass within the collar over time. This interpretation is consistent with the fact that visual stimulation, which is known to trigger PN bouton pruning (Scholl et al. 2014; Stieb et al. 2010), is comparatively low in the hive during the winter months. It is possible that bees experienced their initial sensory exposure to light during their first 2 months in fall, when mild weather allowed for occasional flights. Following this period, PN bouton numbers appear to be maintained at a constant level, allowing functional flexibility under both dark and light conditions when warmer temperatures permit spontaneous trips outside. These findings resemble those of Scholl et al. (2014), who exposed bees of different ages to controlled light cycles over 3 days. In that study, light exposure resulted in a significant reduction of visual PN bouton density in summer bees but had a much milder effect in winter bees, suggesting reduced sensitivity to isolated visual stimuli during overwintering. However, it is remarkable that winter bees maintain bouton numbers despite high energy costs during overwintering.

In contrast, PN boutons in the lip region of winter bees in our study showed both a continuous decrease in density and clear evidence of age‐related pruning (Figure 3B,C). Given that olfactory stimuli are consistently present inside the hive, this likely represents adaptation to the sensory environment. Such pruning may reflect a form of homeostatic plasticity—a compensatory mechanism that helps maintain stable neuronal function following non‐associative sensory exposure (discussed in Groh and Rössler 2020). Supporting this interpretation, a study by Behrends and Scheiner (2010) found that winter bees perform equally well as summer bees in associative olfactory and tactile learning and discrimination tasks, indicating that their cognitive abilities are preserved throughout the winter. This suggests that the MB calyces provide sophisticated adaptations in the interplay of PN boutons and KCs to maintain full capacity to perform all required tasks in the extended overwintering period without diminishing their cognitive abilities. This interpretation is consistent with the unique behavioral demands honeybees face during the overwintering period. The formation and regulation of the winter cluster—including its thermoregulatory dynamics, spatial movement within the hive and the possible rotation of individual bees between the cluster core and its periphery (as observed in honeybee swarms; Heinrich 1981)—likely constitute a highly complex and coordinated process. Although the mechanisms are not fully understood yet, it is plausible that this process is guided by a combination of different sensory cues, such as olfactory and tactile ones. Thus, it appears reasonable that bees retain their cognitive abilities to fulfill these sophisticated demands.

In addition to PN bouton pruning in the lip region, synapsin labeling also suggested a substantial increase in bouton volume over the bees’ 6‐month lifespan (Figure 2I–L). Electron‐microscopic studies have shown that foraging bees and ants possess significantly larger PN boutons than nurses, with increased postsynaptic connectivity (Groh et al. 2012; Seid et al. 2005; Seid and Wehner 2009). Whether a similar or even greater increase occurred in our study cannot be determined, as synapsin immunostaining provides only an indirect measure of bouton morphology. Nevertheless, as olfactory senses are permanently addressed inside the hive, it is highly plausible that olfactory PN boutons undergo considerable structural remodeling during winter, ensuring optimal adaptation to the prevailing sensory environment.

**FIGURE 5:**
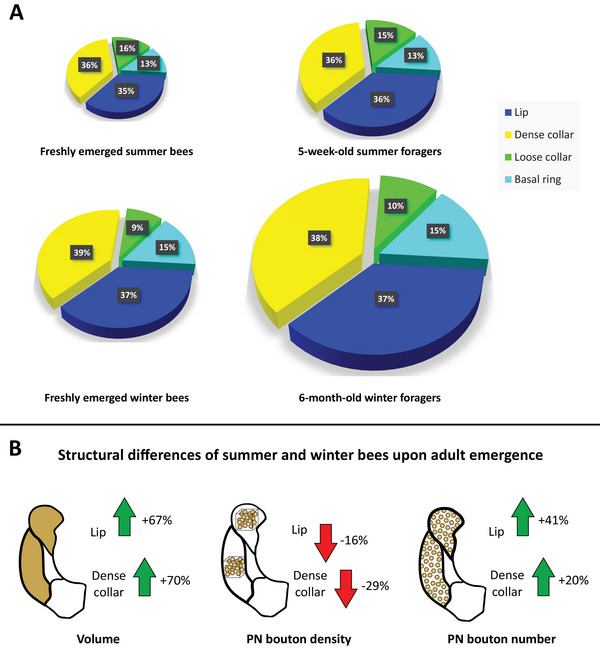
Comparison of structural MB‐calyx plasticity between winter and summer bees at equivalent behavioral stages. (A) Relative changes in MB‐calyx volume of freshly emerged and foraging winter and summer bees. Individual sections of the pie charts reflect the relative proportions of lip (blue), dense collar (yellow), loose collar (green), and basal ring (cyan) in relation to the entire calyx volume. Diameters of the pie charts align with differences in calyx volume. In both summer (upper half) and winter bees (lower half), the relative proportions of individual calyx compartments remain stable over time. Both honeybee phenotypes exhibit a significant increase in calyx volume between freshly emerged (left) and forager bees (right), but in direct comparison, winter bee calyces are substantially larger in both developmental stages. (B) Differences in MB‐calyx structure of winter and summer bees upon adult emergence. The green and red arrows indicate the direction of differences. Freshly emerged winter bees exhibit a 67% higher lip volume and 70% higher dense collar volume than freshly emerged summer bees (left), while densities of PN boutons in lip and dense collar of winter bees are 16% and 29% lower, respectively (middle). Consistently, the combination of both parameters reveals a substantially higher absolute number of PN boutons in lip (41%) and dense collar (20%) of winter bees (right). All data on summer bees are taken from Groh et al. (2012).

While all these results point to similar developmental trajectories of winter and summer bees, the direct comparison of both worker phenotypes, however, reveals a striking disparity in the absolute magnitude of MB‐calyx expansion (Figure 5). While relative growth rates during early adult development and after the onset of foraging are consistent across studies in summer and winter bees (∼42%–50% increase between 1‐day‐old bees and foragers; Farris et al. 2001; Groh et al. 2012), absolute MB‐calyx volumes differ markedly. Considering two studies that have used the same method of synapsin‐based 3D quantifications, we find that 6‐month‐old winter foragers have 55%–68% larger calyces than ∼5‐week‐old summer foragers shown in Muenz et al. (2015) and Groh et al. 2012 (Figure 5A). A direct comparison with data from Muenz et al. (2015), however, proves difficult due to inconsistency in individual experience. Summer foragers from this study did not exhibit a foraging‐associated increase of MB‐calyx volume resulting in a net increase of only 27% between freshly emerged and forager bees, which suggests that they had not yet engaged in foraging long enough to elicit structural changes as suggested by Farris et al. (2001) and Ismail et al. (2006). Therefore, we compared our data in a more detailed analysis with the data by Groh et al. 2012 (Figure 5), where summer bees exhibited similar MB‐calyx growth rates as winter bees. The discrepancy in absolute calyx volumes may result from the fact that winter bees in our study already exhibited larger MB‐calyx volumes upon adult emergence (67% larger lip volume and 70% larger dense collar volume than summer bees from Groh et al. 2012; Figure 5B, left). This corresponded with a lower density of PN boutons in both calyx regions of winter bees, and, due to the enormous difference in calyx volume, higher extrapolated numbers of PN boutons in winter bees (Figure 5B). How do these differences come about? Winter bees develop under conditions of low resource availability but also reduced colony density and absence of brood rearing. It is known that the properties of pollen fed during the larval phase affect the development of the hypopharyngeal glands and fat bodies and thus have a strong effect on potential longevity (Maurizio and Hodges 1950). It seems likely that winter bee destined larvae receive a different quality of nutrition, either due to different types of floral resources, shifted proportions of pollen/nectar ratio, or the amount of food given to individual larvae. Whether these differences in feeding may alter the brain size sustainably, however, is yet to be shown. Alternatively, intercolonial variation could explain the divergence in the data. Comparisons across studies with identical quantification methods reveal substantial variation in calyx volumes of newly emerged bees (Cabirol et al. 2017; Groh et al. 2012; Muenz et al. 2015; our own unpublished data on summer bees; Table S1). For instance, lip volume ranged between 3.24×10^6^ µm^3^ and 5.4×10^6^ µm^3^, with our unpublished summer bee data aligning more closely with winter bees than with prior summer bee studies. Given the temporal (and most likely genetic) proximity of our experiments, colony‐specific effects offer alternative explanations. Considering similar MB growth rates during adult life, a higher calyx volume upon adult emergence might thus lead to an overall higher volume later in life. Nevertheless, we cannot discount the possibility of slow but steady age‐related MB growth. Research on ants suggested continuous MB growth—even far beyond natural lifespans (Gronenberg et al. 1996; Kühn‐Bühlmann and Wehner 2006). Furthermore, data on honeybee queens, which can live for several years, also indicate a continuous age‐related expansion of the MB calyces (Groh et al. 2006). A similar mechanism may operate in long‐lived winter bees. Although we detected no continuous volume increase during winter (potentially due to high variance; Figure 3A), a gradual, age‐dependent process could still contribute to cumulative expansion, particularly in consideration of the large disparity between MB‐calyx volumes of summer and winter foragers (Figure 5A).

Considering the substantial variation in MB‐calyx volumes reported between colonies across independent studies, it is important to note that our samples were derived from a single honeybee colony. As such, the findings should be generalized with caution. However, although honeybee colonies are often treated as single units of genetically identical worker bees, we would like to point out that honeybee queens are typically multiply mated—on average with 15 drones, and in some cases up to 59 (Tarpy et al. 2015, 2004). Consequently, workers within the same colony still exhibit considerable genetic diversity and are further shaped by individual experiences in adult life. Nonetheless, future studies including data sampling from multiple colonies will be valuable to assess potential colony‐specific differences in observed patterns.

Collectively, our findings indicate that winter bees and summer bees share similar MB plasticity mechanisms, differing primarily in lifespan due to reduced foraging‐induced senescence in winter bees. Remarkably, winter bees maintain a high degree of neuronal plasticity and cognitive abilities despite considerable energetic costs of sustaining neuronal networks. However, winter bees also exhibit distinct physiological adaptations, such as a higher fat body mass with larger fat body cells, elevated vitellogenin levels, upregulation of telomerase activity, increased rate of DNA replication in the fat body, and enhanced levels of adenosine monophosphate and oxidized nicotinamide adenine dinucleotide (Fluri et al. 1977; Fluri and Bogdanov 1987; Koubová et al. 2021; Lee et al. 2022). Together, these adaptations are presumed to promote the exceptional longevity of winter bees and may help preserve the function of energetically demanding neuronal networks throughout the extended overwintering period (for reviews, see Amdam and Omholt 2002; Münch and Amdam 2010). Vitellogenin, which naturally accumulates in winter bees due to the absence of brood rearing and also inhibits juvenile hormone synthesis, appears to play a key role for winter survival (Fluri et al. 1982, 1977; discussed in Amdam and Omholt 2002; Münch and Amdam 2010). A study by Münch et al. (2015) has demonstrated that vitellogenin—which likely reduces oxidative stress and enhances immunity (Corona et al. 2007; Seehuus et al. 2006; discussed in Koubová et al. 2021)—is also localized in the brain of winter bees, suggesting it might protect the brain from aging effects.

Interestingly, revisiting our own data from an earlier study revealed that a novel rod‐like structure detected in the KC nuclei of honeybee workers and drones (Figure 5C–F in Hurd et al. 2021) and—less pronounced—in queens and late pupal stages (Figures 4C,G and 5A,B in Hurd et al. 2021) can also be found in winter bees (Figure 1 in Hurd et al. 2021 is based on samples from winter bees, whereas those in Figures 4 and 5 were from summer bees). While the function and biological role of this honeybee‐specific structure are still unknown, a potential involvement in neuronal maturation and KC plasticity is presumed, which supports the idea that winter and summer bees share common underlying mechanisms of plasticity.

## 5 Conclusions

Our study presents the first 3D analysis of age‐ and task‐related structural plasticity in winter bee MB calyces and their synaptic microcircuits. We demonstrate that winter bees retain an impressive degree of neuronal plasticity throughout their extended lifespans during wintertime which strongly correlates with their distinct behavioral flexibility. Contrary to our initial expectations, the neuronal network was not significantly reduced to lower metabolic costs during the highly energy‐demanding period of the colony cycle. Volumetric expansion in all MB‐calyx compartments at the onset and after winter suggests that neuronal maturation is still active and in progression despite the bees’ advanced age. Collectively, we conclude that winter bees exhibit similar neuronal capacities and structural predispositions as their summer counterparts despite their distinct life histories and advanced age. Both phenotypes follow similar patterns of neuronal plasticity in response to intrinsic maturation and extrinsic triggers, such as foraging tasks. However, winter bees shift a substantial part of this plasticity to a later stage in life without facing trade‐offs between neuronal maturation and cognitive abilities. Future approaches might benefit from focusing on the ultrastructural plasticity using electron‐microscopic or high spatial resolution techniques (Kraft et al. 2023) to further unravel the cellular mechanisms behind the drastic volume expansion and to assess the synaptic rewiring at microglomerular complexes.

## Ethics Statement

Our protocols comply with standard welfare practice in our field. Since our experiments were performed with insects, no ethical approval was required for this study.

## Conflicts of Interest

The authors declare no conflicts of interest.

## Supporting information




**Supplementary Table**: dneu23006‐sup‐0001‐TableS1.pdf

## Data Availability

All relevant data are included in the manuscript. Raw data can be made available by the author upon reasonable request.
