# Supplementary material for: Neuronal Plasticity in the Mushroom Bodies of Winter Bees Is Retained Despite Substantially Advanced Age
Source: Dev Neurobiol. 2025 Sep 27;85(4):e23006. doi: 10.1002/dneu.23006 (PMC12476488; doi:10.1002/dneu.23006)
Supplement: Supplementary file 1 — Supplementary Table: dneu23006‐sup‐0001‐TableS1.pdf [file DNEU-85-0-s001.pdf]

Table S1 Comparison of MB-calyx volume of freshly emerged honeybee workers across different studies[1]

**Reference**	**Mean volume [µm³ × 10⁶]**				
	Lip	Dense collar	Loose collar	Basal ring	Total calyx
**This study**	5.40	5.62	1.29	2.21	14.52
**Muenz et al., *Dev Neurobiol*, 2015**	4.00	3.94	1.67	1.47	11.08
**Groh et al., *J Comp Neurol*, 2012**	3.24	3.31	1.42	1.18	9.15
**Cabirol et al., *Learn Mem*, 2017**	~3.80[2]	~3.10[2]	-	-	-
**Own unpublished data**	4.83	4.56	1.37	1.93	12.69

[1]All data are sampled from studies using anti-synapsin immunolabeling-based 3D quantifications in whole-mount brains to ensure comparability.

[2]Values are estimates based on the median of boxplot graphs in the publication as no absolute values are stated in the text.
